# Covid-19 and pathways to health inequities for families in a socioeconomically disadvantaged area of Sweden – qualitative analysis of home visitors’ observations

**DOI:** 10.1186/s12939-021-01556-6

**Published:** 2021-09-26

**Authors:** Madelene Barboza, Anneli Marttila, Bo Burström, Asli Kulane

**Affiliations:** 1 Department of Global Public Health, Equity and Health Policy Research Group, Karolinska Institutet, 17177 Stockholm, Sweden; 2 Region Stockholm, Centre for Epidemiology and Community Medicine, Box 45436, 104 31 Stockholm, Sweden

**Keywords:** Health inequities, Covid-19, Home visiting, Early Childhood Development, Social determinants of health, Qualitative study, Reflexive Thematic Analysis

## Abstract

**Background:**

Lack of control over life situations is an important social determinant that may negatively affect parental and child health. This study took place in an area of Stockholm, Sweden with high indications of socioeconomic disadvantage, a large part of the population with foreign background, as well as higher levels of poor health than the county average. It investigated staff perceptions of pathways from situations of low control, potentially leading to health inequities, affecting families enrolled in an early childhood home visiting programme during the Covid-19 pandemic.

**Methods:**

Semi-structured interviews were carried out with 23 child health care nurses and parental advisors working in a home visiting programme. The data was analysed using Reflexive Thematic Analysis.

**Results:**

The analysis resulted in five pathways on two explanatory levels, affecting parents’ health and parenting capacity and children’s health and well-being, potentially damaging health and leading to health inequities. The first four pathways related to control at the personal explanatory level: Families facing instability and insecurity; Caring for children in crowded and poor housing conditions; Experiencing restricted access to resources; and Parenting with limited social support. The fifth pathway, Living in a segregated society, covered the collective experience of lack of control on community level. The Covid-19 pandemic was observed to negatively affect all pathways and thus potentially aggravate health inequities for this population. The pandemic has also limited the delivery of home visits to the families which creates further barriers in families’ access to resources and increases isolation for parents with already limited social support.

**Conclusions:**

The diversity of pathways connected to health inequities presented in this study highlights the importance of considering this variety of influences when designing interventions for socioeconomically disadvantaged areas. The additional negative consequences of Covid-19 indicate the need for sustainable preventive early childhood interventions for families in such areas. The study also emphasizes the need for further research as well as policy action on possible long-term effects of changing behaviours during the Covid-19 period on child health and health equity.

**Trial registration:**

The study was retrospectively registered (11 August 2016) in the ISRCTN registry (ISRCTN11832097).

**Supplementary Information:**

The online version contains supplementary material available at 10.1186/s12939-021-01556-6.

## Background

The family into which a child is born, the community where it grows up, plays and studies are all aspects which will affect its health [1]. Theories inform of how social factors exert influence on ill-health and health inequities, understanding that interactive layers of social determinants ranging from the macro-level of society to the living and working environment, as well as lifestyle factors, will determine the health of individuals and groups. These socially produced determinants act on populations in systematic ways, producing health inequities that are strongly related to socioeconomic stratification [1–3], where health status follows a social gradient and gradually worsens going down the social scale [1, 4]. The social gradient of health has been confirmed also in Sweden by the Swedish Commission for Equity in Health [5]. In addition, the Swedish Commission presented systematic differences in health between the general population and a number of groups in vulnerable situations, one of which was the foreign-born part of the population who reported worse health than persons born in Sweden [5]. The social determinants of health affect children from the start of life and therefore early childhood development (ECD) interventions are considered to be important tools to break the cycles of health inequity [6–9].

The concept of power has also been given attention with regards to health inequities. The World Health Organization’s (WHO) Commission of Social Determinants of Health (CSDH) stated that inequities in health are caused by social conditions with unequal distribution of resources as well as power between different groups, calling for material, political and psychosocial empowerment of people, communities and countries [1]. The relations between power and health have been investigated by Whitehead and colleagues [10] in a cross disciplinary synthesis of theories linking the degree of control experienced in the living environment to the development of health inequities. The study resulted in a number of hypothesised pathways where situations of lack of control could result in ill-health and subsequent inequities in health.

With regards to how the Covid-19 pandemic has affected populations, initial suppositions that the virus would not discriminate between people were soon disproven and it has become clear that the inhabitants of socioeconomically disadvantaged areas run higher risks of becoming infected as well as dying [11–13]. A study in Sweden also confirmed the influence of the social determinants of health and that low income, low education and having migrated from a low- or middle-income country all predicted higher risk of dying in Covid-19 [14].

From the knowledge areas of social determinants of health and health inequities, as well as ECD, there is a consistent message calling for more research to improve understanding of how and why health inequities are produced and how they can best be prevented through interventions. Whitehead et al., in their study, called for empirical research into the causal mechanisms and pathways to health inequities in order to produce findings that may inform policy [10].The CSDH as well as the authors of the Nurturing Care framework for ECD and the Lancet series of Child Development stated that there is still a weak knowledge base on effective early childhood strategies for health equity, recommending more research and evaluation [1, 7, 15]. To our knowledge, no studies have yet been done with focus on the experience of Covid-19 among families with young children in socioeconomically disadvantaged areas from the perspective of health inequities.

The aim of this study was thus to investigate the pathways of health inequities among the families participating in an equity-based extended home visiting programme in a socioeconomically disadvantaged area of Stockholm, Sweden, with specific consideration to the consequences of the Covid-19 pandemic on these pathways as well as the home visiting programme in this context.

## Methods

### Theoretical framework

This study was guided by the concept of pathways to health inequities presented by Whitehead et al. [10] where theories of the notion of lack of power or control over one’s life that affects health, were investigated within various academic fields. The theories were grouped into a number of pathways on three explanatory levels. The micro/personal level was composed of situations where social position determines the resources that are available for people to control their living situation as well as important life decisions. The meso/community level focused on collective control (or lack of) in the community to influence conditions in the neighbourhood. The macro/societal level include processes of social transitions that affect life control over their lives of whole societies or cultural orientation towards large population groups (for example gender bias). On the three levels, different situations were outlined, detailing the pathways of how they might affect health on individual or group levels.

### Study setting

The area of Rinkeby, located in the northwest of Stockholm, Sweden, can be considered an example of social and health inequities, presenting socioeconomic and health indicators that are among the worst in the Stockholm region [16, 17]. Its population is 61% foreign born and another 30% are born in Sweden with two foreign-born parents [18], which increases vulnerability to health inequities [5].

An extended early childhood home visiting programme was initiated in 2013 in the area, in an effort to reduce the growing health inequities in the capital region [16]. The intervention is a collaboration between the local child health care (CHC) centre and the preventive social services, carried out by teams of CHC nurses and trained social workers, called parental advisors. Embedded within the National CHC programme, which in the Stockholm region provides one home visit to all parents when the baby is born, the intervention delivers five additional home visits over 15 months. It is offered to all fist-time parents at Rinkeby CHC centre [19]. The home visiting programme in Rinkeby offers content in line with the Nurturing care framework for ECD [7, 15], encompassing the provision of good health and nutrition as well as stimulus for early learning and providing a safe and secure environment, with focus on the key role of parents in providing nurturing care practices. The intervention aims to strengthen parenting skills and promote health and well-being for both parents and child [19, 20]. The home visiting staff adopt a health promoting and strengthening perspective, adjusting the intervention to each family’s situation. The parental advisors also act on families’ extra needs and challenges by offering additional psychosocial support and helping them to access further services and resources [21]. The programme is well-evaluated by staff and parents and acceptance rates are above 90% [22].

The families in Rinkeby’s home visiting programme present considerable diversity on several demographic characteristics. The mothers participating in the first programme phase and evaluation, 2013–2016, reported coming from 30 different countries and, while around half of the group had lived in Sweden for 3 years or less, 10% had resided more than 10 years in the country. Levels of education varied widely, with around 40% having less than 9 years of schooling, but a substantial part of almost one third of the mothers having 13 or more years of education. One third were living without the child’s father or a partner. About two thirds were living in rented apartments, while the rest were temporary lodgers (30%) or staying with relatives (10%) [23]. These figures may indicate a large plurality of situations which could also potentially influence health and health inequities among the families in Rinkeby.

During 2020, the first Covid-19 wave hit hard in the Rinkeby community [24]. In this situation the home visiting programme was not able to offer support to Rinkeby parents to the same degree as before. The home visits were interrupted for parts of the year due to risk assessment, resulting in fewer visits on the whole. During some periods visits were substituted by meetings at the CHC centre with only one parent present. There were also differences in the risk assessment between the CHC and preventive social services, leading to the parental advisors being absent from the visits during a considerable part of the year while the CHC nurses carried on the programme by themselves. The CHC centre in Rinkeby has stayed open during the whole period, but restricting the number of family members present. The preventive social services have maintained opening for urgent matters while directing most activities on-line. Both organisations have participated in disseminating Covid-19 information to the population in the area, but they have not been assigned any other Covid-19 related duties.

### Study design

The current study is part of a larger on-going mixed-methods evaluation that has been following the development and implementation of the programme since its beginning in 2013. More details on the evaluation design can be found elsewhere [19] and two reports presenting results are available in Swedish [22, 23].

### Data collection

The investigation was carried out through semi-structured interviews with the CHC nurses and parental advisors who delivered the home visits in the Rinkeby extended home visiting programme.

The study included all home visiting staff that had worked or were still working in the programme since its start in 2013, a total of 26 professionals, of which seven were parental advisors and 19 CHC nurses (see Table 1). All seven parental advisors were first interviewed in presence by the first author between June and Oct 2019. Part of the data collected in these interviews was also used in another study [20]. Interviews lasted 90–120 min. Additional interviews were then conducted in Dec 2020-Jan 2021 with four of the seven parental advisors who had been working in the programme during the Covid-19 outbreak in 2020, using an additional interview guide to specifically investigate their understanding of the families’ situations and the functioning of the programme during this period. These interviews lasted 32–40 min. All parental advisors were female and, except for one who was a family therapist, they were all trained social workers. Their work experience in the programme ranged from 1–5 years. From the group of 19 CHC nurses who had worked in the programme during various periods and length of time from 2013, 16 agreed to participate in the interviews, two declined giving reasons of lack of time and not feeling capable of contributing with relevant information, while one did not reply to contact attempts. The interviews with nurses were carried out in Dec 2020-Jan 2021 and lasted between 21 and 53 min. All nurses were female and trained specialist nurses in CHC or primary health care. The nurses’ work experience in the programme ranged from 0.5–7.5 years and eight of them had worked during the Covid-19 outbreak in 2020. The interview guide (Additional file 1) contained additional specific questions related to the Covid-19 outbreak which were asked to those eight nurses.

All interviews during 2020–2021 were carried out by the first author via a video-conference app with sound and visual recording. They were transcribed by the first author.

**Table 1:** Overview of interviews with home visiting staff

**Professionals**	**Interview participation**		**Nr. of interviews**
3 Parental advisors	Interview 2019		3
4 Parental advisors	First interview 2019	Additional interview 2020/21 regarding work during Covid-19	8
8 CHC nurses	Interview 2020/21		8
8 CHC nurses	Interview 2020/21 including additional questions on work during Covid-19		8
**Total professionals = 23**			**Total interviews = 27**

The data collection was guided by the notion of pathways of Whitehead et al. [10], through interview questions where the professionals were asked to describe the living situations of the families in the home visiting program, reflect on what types of challenges the families encounter and how this influences their lives, health and parenting. Similarly, the interviews investigated the consequences of Covid-19 on families’ life situations.

### Data analysis

The data was analysed using Reflexive Thematic Analysis following the six-phase process delineated by Braun et al. [25–27]. Reflexive Thematic Analysis was considered suitable due to its objective to produce a consistent interpretation of data through the identification of patterns of meaning that are shared within the set of data. While the method recognizes the researcher’s active reflexive engagement in the analysis where the subjectivity is considered a resource, it is also flexible to the use of different guiding theories in the analytic process [25–27]. All transcripts were read through and notes were taken in order to obtain familiarisation. Systematic coding of the first three interviews was carried out by the first author and reviewed and discussed with the fourth author. Subsequent coding was then done for all remaining interviews. The development of themes, following the data coding, was then directed by the attempt to identify different types and patterns of lack of control experienced by the families as well as understanding their organisation in pathways, guided by the ideas of Whitehead et al. [10]. This process was led by the first author and continuously presented and discussed with the group of authors. The initial themes were presented in research group and departmental meetings which contributed to the revision, definition and naming of final themes, as well as their division into the explanatory levels proposed by Whitehead et al. [10]. These were discussed and the draft finalised with all authors. Coding and analysis were supported by the computer software Open Code 4.0.

Consideration also needed to be taken of the fact that the study was done in a named area with a relatively small group of professionals, and thus total anonymity could not be assumed despite the removal of names from all data. Therefore, the analysis was kept on a group level, care was taken to avoid giving examples or presenting quotes of a more personal nature, and quotes were only identified by profession.

### Trustworthiness

To ensure credibility of the study, all professionals from the home visiting programme in Rinkeby were included in the study population. The initial intention to also interview parents was abandoned after a period of unsuccessful attempts of recruitment during November 2020. This decision was taken after discussions and on the advice of the management of the CHC centre. Further, all authors were actively involved in the analysis process, and the preliminary results were shared with co-researchers and experts. High work pressure for the CHC nurses, especially during the Covid-19 pandemic, made it unfeasible to request any feed-back from the professionals on the findings of the study during the analysis process. In order to guarantee dependability, the same general questions were used in all interviews, adding specific questions related to Covid-19, for the relevant part of professionals. Comparisons were carried out between interviews from 2019 and from 2020/21, and no significant discrepancies in questions or content were identified.

## Results

**Fig. 1:**
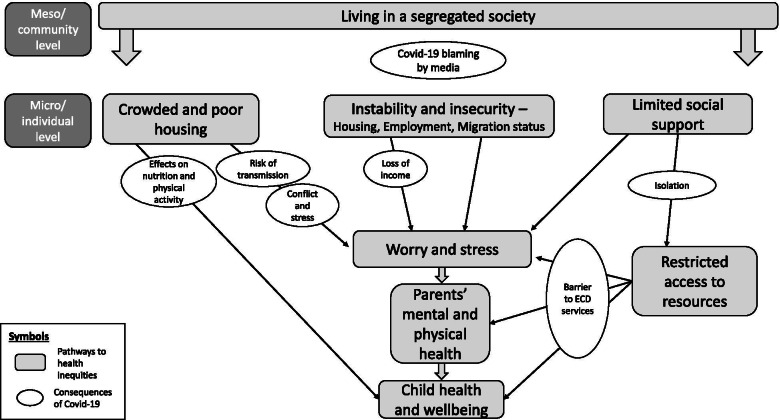
Pathways from low control to potential health inequities

The Reflexive Thematic Analysis of the interviews with home visiting staff resulted in five pathways from situations of low control in the living environment which may affect parents’ health and parenting capacity and children’s health and well-being, potentially leading to health inequities. The first four pathways relate to control at the micro/personal explanatory level: Families facing instability and insecurity; Caring for children in crowded and poor housing conditions; Experiencing restricted access to resources; and Parenting with limited social support. The fifth pathway, Living in a segregated society, regards the meso/community level. Negative influences of Covid-19 were identified within all five pathways. The consequences of the pandemic on home visiting could be seen as creating new barriers which primarily affected the pathways of Experiencing restricted access to resources; and Parenting with limited social support. The pathways are presented through the following text and citations and are also illustrated in Fig. 1.

### Families facing instability and insecurity

The most common situations related to lack of power or control, reported by the home visitors, concerned some families’ experiences of housing and economic instability. Insecure living conditions are common with subletting apartments and rooms without formal contracts, leading to frequent moves and constant threats of sudden evictions.

*“You don’t have a first-hand contract. Maybe you live second-hand, third-hand, as a lodger. And that, in itself, you don’t have a safe place where you feel is home. And that causes stress in the parenting.” (CHC nurse).*

Economic insecurity is also a frequent concern, where families often depend on one income only. Access to the formal job market is a challenge, and parents are forced to accept informal work and sometimes juggling several jobs, with long work hours as a result. An additional aspect of instability and insecurity is experienced by those families living without formal residency permit in the country, as illegal immigrants or caught in the bureaucracy awaiting decisions on refugee status. All these different situations are recognized by the home visitors as a pathway leading to chronic stress which may affect parents’ mental and physical health, their parenting capacity and consequently also the health and development of their children.

*“I think it affects their psychological well-being a lot. I notice that some of them really don’t feel well. If there is pressure of only staying in the apartment for a few months, that can of course influence mental health. Economy as well, or other insecure situations. If you don’t have documents for example, there is a big insecurity and you can’t really cope.” (Parental advisor).*

With concern to this pathway, Covid-19 has represented an additional source of worry on top of the instability already present. Many of the parents have jobs with high risk of exposure to the virus, which has caused concern. Some concrete consequences have also been noted on employment insecurity, with parents having lost jobs, but also a general increase in worry and stress over the possibility to lose their incomes.

*“They work in healthcare professions, taxi, busdrivers, so it has hit very hard here too. I have several mothers who have talked about the financial aspects, maybe the father has worked and now lost his job. So it feels like it has hit very hard on many dimensions here.” (CHC nurse).*

### Caring for children in crowded and poor housing conditions

Closely connected to the economic and housing insecurities experienced by some families, are the conditions under which some families are living. The home visitors confirm that it is common for families with numerous children to live in one- or two-bedroom apartments, or several families sharing apartments. Families living in a small room with limited access to kitchen facilities and thus facing difficulties in preparing nutritious food for their child is an example that illustrates how this pathway may have a direct influence on child health and development. Another concrete situation is how parents are not able to ensure the living environment is hazard-free, exposing the children to risks of accidents or risks such as smoking by other tenants. For some of the single mothers, often newly arrived in the country, the only option is a room in short-term accommodations, called “hotel homes”, provided by the social services, where they are exposed to potential stress, feeling discomfort and unsafe when having to share kitchen and bathroom with strange men.

*“Of course it affects, their thoughts are taken up by these things. And I can find it frustrating, that sometimes I can’t even give all advice, if the parent doesn’t really have a place to live and no possibility to cook. […] So, concretely, things get very troublesome for a family with a small child if you have a very spartan living situation, or live with other people where you feel you can’t behave in the way you would otherwise like to.” (CHC nurse).*

Covid-19 has increased the potential negative consequences of this pathway in several ways, first of all through the increased risk of transmission due to crowded living.

*“And this, that no-one talks about how the situation looks like. The housing issue is very important during a pandemic. Anyone knows that if you look at the different pandemics hundreds of years back. What does it look like for the families? It is not uncommon for us to see a family with five children in a one-bedroom apartment. So it hits hard of course. I think the pandemic has both pros and cons because it has really made clear what it really looks like in these areas and what the political decisions have led to.” (CHC nurse).*

The home visiting staff perceive that during the first phase of the pandemic from March to August 2020, many families in Rinkeby isolated themselves in their homes to a higher degree than the population at large. Children were also kept at home although daycare and elementary schools stayed open. The combination of isolation and crowded living conditions was perceived as an increased risk of conflict and stress within the family.

*“There are so many causes for conflicts now, the economy is down, the children are not in day-care, you step on each other’s toes much more easily now. I think it definitely causes more negative things than usually, but that we may see it now is a bit hard to tell. We may not see it in some months, the consequences of all this, that it doesn’t show up on the bill immediately.” (CHC nurse).*

Another very clear related effect on child health has been noted by the CHC nurses, where keeping children isolated in small spaces has led to higher intake of unhealthy food and less physical activity and a consequent unprecedented increase in child overweight and obesity in the area.

*“Those who have had a baby, they keep the siblings at home, which means a very small space to be in. And when you have a newborn, it is hard to go out so you spend more time indoors, more sweets, more TV. So we have seen a great increase in weight on many children. I have never seen weight curves like this in my life.” (CHC nurse).*

### Experiencing restricted access to resources

Within the large diversity of parents in Rinkeby, three groups stand out as more disadvantaged to the home visiting staff: those with very low levels of education, those who do not speak the Swedish language, and those with little knowledge of the functioning of the Swedish society. The parents who find themselves in one or more of these groups experience higher barriers regarding the access to information as well as access to health and social care and other resources in society. The home visiting staff notice this barrier in relation to the families they meet.

*“And that is the biggest challenge, to find some way to try and meet each other and understand each other in both directions, for the families and for us.” (Parental advisor)*

Not speaking the language also makes parents dependent on translation help from friends or relatives or from official interpreters in their contacts with authorities and when using resources such as the home visiting program.

The appearance of Covid-19 has placed further barriers to the access of resources in this pathway. The pandemic has affected the functioning of services aimed at parents with young children such as the home visiting programme, parenting groups, the open day-care, some of which have had to close down during periods. Access to health care has also become more challenging than usual.

*“What I have heard from families is that the health care was already hard for them to reach. They were often sent from one place to another, ‘no we cannot receive you’. It has become harder during Corona, not knowing where to turn. Actually no one knows, but it was even worse for these families, knowing when to seek medical care and how, because you get lost in this big healthcare model.” (CHC nurse).*

Several consequences of Covid-19 on the home visiting programme are also placed within this pathway. The most notable has been the absence of parental advisors in the home visiting team for large parts of the year, which means some parents have not met them at all. The nurses perceive difficulty in introducing some contents that are usually led by the parental advisors, especially regarding violence, and also a decrease in quality in the meeting with families during times of increased need.

*”The whole idea is built on the interplay between CHC nurse, parental advisors and parents, so I feel it is a great loss really. I think it is even more needed now during the pandemic.” (CHC nurse)*

A digital solution has not yet been found to include the parental advisors during the visits, but they are available for individual advice and counselling over the phone or online outside of the visits. However, there is a higher threshold for parents to make this contact when there is no or little initial relation created through the home visiting.

*“To handle the worry I think would have been a great contribution of the parental advisors now. And just to establish the relation because it may be difficult, even when we refer to them when we notice that the parents are very worried, because there is no established contact it is harder to take that step than if they already had a name and face.” (CHC nurse).*

Similarly, the door-opening function to the rest of social services usually performed by the parental advisor has now also become affected. What earlier could have been a physical meeting between parent and social services, supported by parental advisor and interpreter when needed, has now become a cumbersome task where the parents are left on their own to a larger extent.

*“I have contact with a parent, where I believe her contacts with social services for economic support have been more by telephone lately. It is of course much harder for her to make her case over the phone, and then it is really essential that the professionals she is in contact with make sure to book an interpreter. Because it gets even harder. When you have a physical meeting with someone, well you can sort of understand each other. It gets much harder on the phone when you can’t use body language or write something. So I can see that it has become hard for some of them.” (Parental adviser).*

Another significant barrier due to the pandemic is the increased restriction of fathers participating in the home visiting programme. When the encounters were moved from the families’ homes to the CHC centre, restrictions allowing only one parents were introduced, which in practice mostly affected the fathers. This is noted with concern by the staff, considering that the systematic incentives to motivate fathers’ participation in the programme have been given high importance over the years.

The communication issues experienced by families in this pathway have also become aggravated during Covid-19, further limiting their access to information and resources. During the home visits for example, those parents who do not speak Swedish are not allowed to bring someone to help with translation and official interpreters are only available over the phone, something that makes communication significantly more difficult. Similarly, the use of protective equipment by the nurses is a further barrier to communication with parents.

*“During Corona, for those who don’t speak Swedish, it has been very hard. With telephone interpreter, facemask and shield. The shield in itself creates a barrier. And they don’t see my face. Especially the first meeting I feel is less personal and spontaneous. The conversations become more informative, the way the visits are not meant to be.” (CHC nurse).*

### Parenting with limited social support

While many parents in Rinkeby have large extended families and social support network, there are also parents with virtually no one else to count on. One such group are single mothers who arrive in the country on their own, pregnant, with the father abroad. Other groups are mothers where the father is working long hours away from the home, or the father does not take an active role in the child’s life. What they all have in common is the pathway of lack of social support, experiencing isolation and loneliness, which may affect mental and physical health and parenting skills.

*“They were living with another lodger who was smoking a lot, so the whole apartment was smelling of smoke. But they could not change that, and the mother was alone at home during the days and didn’t manage to get down all of those stairs by herself with the children and pram and everything. So it became such an isolation.” (Parental advisor).*

For these parents an important channel for social support is when the home visiting staff guide them to the open day-care, where they can get out of the house, participate in activities and meet other mothers and children. Covid-19 has created new barriers and increased the isolation for these families. During the pandemic the open day-care has opened only for a limited number of visitors, part of the time all activities have been held outdoors, and in periods it has been closed.

*“I think it gets harder and harder now when it is dark and a bit depressing outside. So many people stay at home. It has been a tough year for everyone, you can notice it. And there are quite many lonely parents, they have become even lonelier now. Before we have had the open day care, the family house, different channels to guide them to, but it has been pretty impossible during the pandemic. So they have told us about very much loneliness.” (Parental advisor).*

### Living in a segregated society

Rather than focusing on the life situation of individual families, this pathway regards the collective experience of living in Rinkeby. The home visitors narrate the populations’ perceptions of a structural cutting down of resources in the community, such as the police station, job centre and district administration, all of which contribute to Rinkeby being considered an unsafe and disadvantaged area to live in.

*“As soon as they get a better life situation they move to a neighbouring area or municipality because they feel that Rinkeby is unsafe and they don’t want their children to grow up here.” (CHC nurse)*

Many of the inhabitants are subject to discrimination and there is a perception that the authorities have left Rinkeby to its fate.

*“So what you see is the effect of the dismantling of society that has led to it looking like this in this area. And one should look up and see how the schools look, how the environment looks, how the job market looks. Many people tell of racism in society, like, should you need to change your name to a Swedish name to get a job? I see that part too. That they think you should lift your eyes a little, and it is not about totally helpless individuals. What they want is to get equal citizen rights. That is what I see.” (CHC nurse).*

This narrative also contains another aspect of segregation which is the experience of lack of cohesion, when families’ own backgrounds and cultural values do not coincide with those of the Swedish system.

*“And to learn to live in a new country with children, where there are different habits and ways than what you used to do in the home country. Things that worked there and then you have to adjust to a new way that you may not agree with or feel at home with and everything becomes different, new and strange.” (CHC nurse).*

The staff recognize that these elements interact with the other pathways, for example by increasing barriers to access resources. A concrete example is how mistrust in the system makes families suspicious and afraid of accessing social services.

*“Sometimes they are afraid, ‘The social services, why do they want to come in? Why are they here? Is it something special or is it because we are bad parents?’, that is how it is interpreted.” (CHC nurse).*

Covid-19 has affected this pathway in a double sense. The community was hit hard in the first wave and many of its inhabitants were forced to higher risks of exposure to transmission in work and family life. Still, the home visiting staff believe that the families in Rinkeby were well informed of the situation and adhered to a, in many cases, stricter social distancing protocol than that prescribed by the Swedish public health agency. Despite this, the community was depicted in media in what could be perceived as a blaming manner.

*“There were newspaper articles about it and the reasons why [transmission was higher]. I think some people felt it as groundless criticism, as if it was due to them not following restrictions, that they were illiterate and hadn’t taken in the information. So I think a lot of people were quite sad about that. Because it was definitely not the way it was perceived in the community, but rather that many people were very aware of what was happening.*“ *(CHC nurse).*

## Discussion

The interviews with parental advisors and CHC nurses in the Rinkeby extended home visiting programme were analysed with the aim of identifying situations where families from the programme experienced lack of control over their living environment which affected the health and wellbeing of parents and/or children, and could potentially create or aggravate inequities in health. The analysis generated four pathways at the micro/personal explanatory level and one pathway at the meso/community level, which all corresponded well to the possible pathways proposed in the synthesis of theories by Whitehead et al. [10]. The micro level pathways in our study all referred to “actual” control pathways as opposed to “perceived” control pathways, also introduced in the synthesis of theories, where lack of power and control is a socially constructed belief. These four “actual” control pathways included the elements of “money, power, information, prestige, environment and uncertainty about the future” proposed by Whitehead et al. ([10], p.54). Similarly to the synthesis of theories, the pathways in our study lead to “chronic stress response” and “exposure to health damaging living environments” ([10], p.54), the former is connected to consequences on parents’ health in our study while the latter was reported as more directly leading to possible consequences on the children’s health. Our study also indicated how parents’ mental health directly affected children’s health and wellbeing. Differently to the synthesis of theories, no situations of “health damaging” behaviour, where lack of control leads to, for example, drug or alcohol use, were observed by the interviewed home visitors in our study. The meso/community level presented many similar elements to the synthesis of theories, such as “segregation, safety, collective mistrust and low investment in public services” ([10], p.56). In our study it was expressed in terms of elements in the context which interact with and influence the microlevel pathways, rather than being pronounced as a separate pathway in itself leading to health inequities. This analysis is also in agreement with the observation of Whitehead et al. that the different levels of pathways should not be considered separately but as parts of a complex interrelation of social determinants of health [10].

With regards to Covid-19 and health inequalities, Bambra et al. [28], already in April 2020, predicted several mechanisms through which the pandemic would have more serious impacts on the most socio-economically disadvantaged groups in societies. These included higher infection rates and risk of death due to already higher rates of non-communicable diseases, high risk working- and living-conditions, higher susceptibility due to adverse psychosocial conditions and restricted access to health care. Further, the authors foresaw that lockdown measures would hit harder in these groups [28]. Our analysis similarly observed a number of situations where the pandemic negatively interacted within all the pathways to health inequities, involving situations of higher exposure to Covid-19 among the families, less possibility of social distancing, as well as several negative impacts on parents and children resulting from increased isolation at home, barriers to service access and Covid-19 blaming by media.

Looking closer at the pathways, the most commonly discussed determinant in our study was housing, influencing health inequities in two distinct manners, via crowded and poor housing conditions, as well as by representing instability and insecurity in the lives of the families. In two other studies, similar pathways of housing insecurity [29] and poor conditions [29, 30] leading to ill-health were identified by people themselves experiencing these conditions. Like in our research, both studies included poverty and employment insecurity in the pathways, also discussing the negative experience of insecurity and instability caused by these material-structural aspects and how they, in themselves, are recognized as affecting health [29, 30].

Several consequences of Covid-19 were found in our study in relation to crowded and poor living conditions. They correspond to the higher risk of transmission of Covid-19 due to difficulties in keeping social distancing in crowded living conditions, discussed by other authors [11, 13, 28, 31], as well as the potential increase in stress and conflict [28]. In addition, our study indicated already detectable effects in terms of considerable weight gain in children, something which may lead to future ill-health and health inequities. These findings suggest the need for further research as well as policy action on possible long-term consequences of changing behaviours during the Covid-19 period on child health and health equity.

The community level pathway in our study brought perceptions of experiences of segregation, discrimination, and a sense of loss of cohesion, while welfare resources in the community have been partly dismantled. The accounts of experiencing unsafety for the children in the area and wanting to move away from the community relates well to examples of stigma experiences and coping strategies in the framework of spatial stigma leading to health inequities by Halliday et al. [32]. The authors also recognize the intersectionality between spatial stigma and other channels for discrimination such as class, gender and ethnicity [32], similarly to the mention in our study of exclusion from the job market due to ethnicity. The accounts in our study of media blaming the community for the high Covid-19 spread is also a clear example of a practice that drives the spatial stigma in the pathway to health inequities [32]. Although the community level pathways to health inequities may not be the one first identified by practitioners and the population itself, they enhance our understanding of the critical need to consider the complex interrelation of the social determinants on all levels without forgetting or ignoring the structural determinants and only focus on those more visible on the individual level. In recent years British researchers have reinforced the argument that in order to combat socio economic health inequities it is necessary to consider how macro political and economic structures influence both the communities/place and the health of the individuals living there [33–35], as well as increasing understanding of the mechanisms of power and control on all levels in order to promote community empowerment in favour of health equity [34, 35]. Mackenzie et al. demonstrated that policymakers and practitioners tend to underplay the role of power and politics in favour of material and behavioural explanations in their assessments of the problem of health inequities [36]. This points to the need of promoting the introduction of analyses of effects of structural economic, political and power factors, not only for decision-makers but also at practitioner level in interventions with the aim of decreasing health inequities in socioeconomically disadvantaged communities.

The pathway of Restricted access to resources in our study was connected to education level, but it also included other groups such as those families who have recently arrived in the country, or those who may have high education but do not speak Swedish. Similar findings are presented in a study of home visiting families’ needs and challenges from the United States [37] where the focus was on immigrant populations, indicating that this pathway is of higher relevance in communities with higher levels of migrants. The authors found that language, social and physical isolation, economic hardship, citizenship status and mistrust of formal systems acted as barriers to access to healthcare and social services [37]. While the elements are very similar to the ones found in our study, the organization of the pathways differed. Notably, citizenship status and economic hardship were not directly related to access to services in our study, which most probably mirrors the differences in healthcare and service organization in the two countries.

Although the Swedish government has not imposed any general lockdown in response to the Covid-19 pandemic, and overall less restrictive measures compared to many other countries [38], it is still possible to observe considerable negative influence of Covid-19 in terms of creating further or even higher barriers to resources and services. This is well exemplified through the restricted delivery of Rinkeby’s home visiting programme during 2020, where the partial interruptions of the programme due to risk assessments led to fewer visits on the whole, but also the closing of an important channel for families to reach other services, such as social services and the open day-care. Studies of Covid-19 and health inequalities, so far seem to have focused principally on the consequences in terms of barriers to healthcare access [12, 28, 39]. This aspect was also mentioned in our study, but with regards to the CHC it is contradicted by a report from March–May 2020 of the Regional CHC Programme in Stockholm, which assessed that overall, the CHC services had been maintained to a large degree during the first wave of the pandemic [40]. It seems however, that the longer-term consequences of barriers to accessing preventive services such as home visiting, parenting groups, open day-care or other resources still need to be given attention in research. Our study indicates the increase of these barriers due to Covid-19 and the possible aggravation of health inequities as a consequence, and thus reinforces the importance of ensuring the maintenance of preventive services for families in socioeconomically disadvantaged areas. In addition, the restrictions of fathers’ participation in preventive services and possible long-term influences on children and families need to be followed up.

The final pathway, which also relates to barriers in service access, concerns Parenting with limited social support, where the common denominator is the experience of isolation and loneliness which create stress and worry in the parents. The study observed that Covid-19 has increased levels of isolation, at the same time as the already discussed decrease in service offerings has inflicted higher barriers. The national CHC services reach virtually all families with young children [17] and the CHC centre and preventive social services in Rinkeby have so far been successful in engaging over 90% of the first-time parents in the additional services of the extended home visiting programme [22]. By presenting channels for social networking and offering psychosocial support when there is need, the parental advisor has a key role in reaching the isolated parents that may otherwise be invisible to society [21]. The importance of home visiting in engaging families that may be “hard to reach” by other services has been noted by other studies [37, 41, 42] as well as the negative impact on health equity when ECD services are unsuccessful in this endeavour [43]. These arguments, again, reinforce the importance of not “losing” the already socially isolated families due to interrupted preventive ECD services during the pandemic.

### Strengths and limitations

Smith et al. argue that knowledge on pathways from socioeconomic disadvantage to health inequities are already well understood and that further research may contribute to increased labelling and stigmatisation of communities [30]. We acknowledge this view as well as the ethical responsibility when executing research into concepts such as “vulnerability, disadvantage, control and power”. However, we also recognize that our research carries importance in contributing to the understanding of the relation between control and pathways to health inequities during the early years of life in a socioeconomically disadvantaged and culturally diverse setting, from the perspective of home visiting and other preventive ECD interventions. The onset of the Covid-19 pandemic, representing a new and unprecedented (in our times) situation of loss of control over many aspects of normal life, reinforced our understanding of the relevance and urgency of this study.

The pandemic, however, impeded the access to families and consequently their participation in the study. We believe this was a limiting factor which may have excluded important views and perspectives that could have contributed largely to the richness and quality of this material. While the study focused on the common patterns/pathways as perceived by the home visitors, we are certain that more extensive research with families would add new levels of understanding of an even larger diversity of individual and communal pathways to health inequities.

## Conclusions

This study has uncovered a diversity of ways in which families living in a socioeconomically disadvantaged area might experience situations in their daily life that can lead to health inequities. This complex understanding of interrelated pathways is of importance to apply in designing interventions targeting health equity in such areas. In this context, the Covid-19 pandemic was observed to add further negative influence within the pathways. The pandemic also had detrimental consequences on the ECD support offered to families through an extended home visiting programme, by limiting content, psychosocial support and restricting access to further resources and services. This indicates the importance for policymakers and managers to ensure sustainable preventive ECD support to parents and children, especially in a time of observed higher needs. Finally, the study highlighted examples of negative consequences on child health due to families’ self-imposed isolation in crowded living conditions. This calls for further research as well as policy action on possible long-term effects of changing behaviours during the Covid-19 period on child health and health equity.

## Supplementary Information

**Additional file 1.** Interview guide for CHC nurses and parental advisors from the Rinkeby extended home visiting programme (translated from Swedish).

## Data Availability

Data sharing is not possible according to Swedish law.

## Abbreviations

CHC: Child health care
CSDH: Commission of Social Determinants of Health
ECD: Early childhood development
