# Supplementary material for: Covid-19 and pathways to health inequities for families in a socioeconomically disadvantaged area of Sweden – qualitative analysis of home visitors’ observations
Source: Int J Equity Health. 2021 Sep 26;20:215. doi: 10.1186/s12939-021-01556-6 (PMC8474881; doi:10.1186/s12939-021-01556-6)
Supplement: Supplementary file 1 — Additional file 1. Interview guide for CHC nurses and parental advisors from the Rinkeby extended home visiting programme (translated from Swedish). [file 12939_2021_1556_MOESM1_ESM.docx]

**Additional file 1. Interview guide CHC nurses and parental advisors from Rinkeby extended home visiting programme** (translated from Swedish)

**Presentation**

1. Would you like to present yourself? How long have you worked/did you work in the home visiting programme? Where did you work before? (Where do you work now?)

**Understanding of families, their needs and resources**

1. How would you describe the families that participate in the programme?
2. What do their life situations look like? What are the principal challenges they experience?
3. Which needs do they present during the home visits?
4. Do the challenges in their life situations influence their parenting? If so, in which ways?
5. Do the challenges affect their own or their child’s health? If so, how?
6. What strengths do the parents possess? What strategies do they use to overcome their challenges?

**Questions 7-17 regarding the Covid-19 pandemic (applied to those CHC nurses and parental advisors working during this period)**

1. What does your work in the programme look like at the moment? How have the home visits functioned since the start of the pandemic?
2. How have the child health care centre’s/preventive social services’ activities on the whole been affected by the pandemic (visits, vaccinations, parenting groups, psychosocial advice and support etc)?
3. How do you experience the pandemic in Rinkeby compared to other areas?
4. What is your view on parents’ awareness of the pandemic and restrictions? Please give examples.
5. How well are advice and restrictions followed?
6. How has life changed for the families since the pandemic started?
7. Have the families’ life situations been affected? How?
8. Does the pandemic affect their parenting capacity? How?
9. Do they present different needs during the home visits compared to before the pandemic? What are the differences?
10. If you consider the needs of the families, how well does the home visiting programme in general respond to them?
11. How well has the programme been able to respond to the needs during the pandemic? What has been lost (if anything)?

**The capacity of the home visiting programme**

1. If you consider the needs of the families, how well does the home visiting programme respond to them? (If not already asked as Q16)
2. What competencies are needed to be a good CHC nurse/parental advisor in the home visiting programme?
3. What are the differences between the two professionals’ roles during the home visits? What is similar?
4. What conditions need to be in place for the home visits to work?
5. How could the programme be improved?
